# The role of IFI16 in regulating PANoptosis and implication in heart diseases

**DOI:** 10.1038/s41420-024-01978-5

**Published:** 2024-05-01

**Authors:** Xindi Chang, Bei Wang, Yingli Zhao, Bing Deng, Ping Liu, Yiru Wang

**Affiliations:** 1 Department of Cardiology, Longhua Hospital, Shanghai University of Traditional Chinese Medicine, 725 South Wan-Ping Road, Shanghai, China; 2 Department of Emergency, Longhua Hospital, Shanghai University of Traditional Chinese Medicine, 725 South Wan-Ping Road, Shanghai, China

**Keywords:** Cardiovascular diseases, Inflammation

## Abstract

Interferon Gamma Inducible Protein 16 (IFI16) belongs to the HIN-200 protein family and is pivotal in immunological responses. Serving as a DNA sensor, IFI16 identifies viral and aberrant DNA, triggering immune and inflammatory responses. It is implicated in diverse cellular death mechanisms, such as pyroptosis, apoptosis, and necroptosis. Notably, these processes are integral to the emergent concept of PANoptosis, which encompasses cellular demise and inflammatory pathways. Current research implies a significant regulatory role for IFI16 in PANoptosis, particularly regarding cardiac pathologies. This review delves into the complex interplay between IFI16 and PANoptosis in heart diseases, including atherosclerosis, myocardial infarction, heart failure, and diabetic cardiomyopathy. It synthesizes evidence of IFI16’s impact on PANoptosis, with the intention of providing novel insights for therapeutic strategies targeting heart diseases.

## Facts

- Pyroptosis, apoptosis, and/or necroptosis by themselves are insufficient to describe PANoptosis, an inflammatory form of programmed cell death mediated by the PANoptosome complex.
- IFI16 and AIM2 are HIN-200 family members that show many of the same functions in biological processes. It has been confirmed that AIM2 plays a central role in PANoptosis. IFI16 is connected to apoptosis and pyroptosis and also has a high expression in STAT3β overexpression cells to enhance necroptosis.
- PANoptosis is involved in heart failure, cancer, sepsis, pulmonary diseases, infectious disease, and so on.

## Open questions

- What is the specific role of IFI16 in the PANoptosis progress?
- How does PANoptosis affect heart disease?
- If we intervene in the PANoptosis progress by regulating IFI16, is it possible to prevent and treat heart diseases?

## Introduction

The Interferon Gamma Inducible Protein 16 (IFI16), a member of the hematopoietic interferon-inducible nuclear (HIN)-200 family, stands as a sentinel at the intersection of immunity [1], inflammation [2], and cell fate decisions [3, 4]. Within the intricate landscape of the immune system, IFI16 has emerged as a multifunctional player with the ability to sense DNA damage, detect viral threats, and regulate inflammatory responses. Its role in orchestrating these processes has led researchers to explore its potential involvement in various disease contexts [5–7]. The significance of various interferon-inducing DNA receptors lies in their capacity to initiate a cascade of immune responses and cellular pathways in response to stressors, ultimately influencing disease outcomes [8]. Its interactions with inflammasome assembly, cytokine release, and cell death pathways are particularly intriguing. This review delves into the emerging understanding of the potential role of IFI16 in heart diseases, with a specific focus on its intricate relationship with PANoptosis – a newly recognized form of pro-inflammatory programmed cell death encompassing the simultaneous activation of pyroptosis, apoptosis, and/or necroptosis, but that cannot be accounted for by any of these three programmed cell death pathways alone [9–11].

Central to this review is the concept of PANoptosis – a phenomenon that weaves together different cell death pathways in a synchronized symphony of cellular responses. PANoptosis is an inflammatory programmed cell death that is mediated by the PANoptosome complex and cannot be characterized by pyroptosis, apoptosis, or necroptosis alone [9]. There are four members IFI16, absent in melanoma 2 (AIM2), IFIX, and myeloid cell nuclear differentiation antigen in the HIN-200 family. Only IFI16 and AIM2 can activate the inflammasome [12, 13]. AIM2 and IFI16 have also shown the other same features, such as cytosolic dsDNA sensors, ASC (apoptosis-associated speck-like protein containing a CARD)-dependent inflammasomes, regulating atherosclerosis, autoimmunity, tumorigenesis, and normal neuronal development [1, 14–19]. However, research shows that IFI16-β, a human mRNA variation of IFI16, inhibits the AIM2 inflammasome [20]. Furthermore, since AIM2 and IFI16 cannot be substituted, they must have different characteristics [21]. The ways in which a host uses AIM2 and IFI16 in diverse ways to fight infections require further explanation. As research highlights the interactions of IFI16 with pyroptosis [4, 22], apoptosis [23, 24], and necroptosis, the question of its potential as a PANoptosis regulator emerges. IFI16’s unique ability to sense danger, modulate immune responses, and engage in cell death regulation suggests its potential to orchestrate PANoptosis outcomes in heart diseases.

In this review, we navigate the intricate connections between IFI16, inflammation, cell death pathways, and heart diseases. By delving into the potential roles of IFI16 in heart pathogenesis, particularly in the context of PANoptosis, we aim to shed light on its multifaceted contributions to disease progression. As the pieces of this puzzle come together, we unveil a new perspective on the intersections of immunity, inflammation, and heart health.

## Functions and regulation of IFI16

IFI16 is characterized by the presence of two HIN domains and a pyrin domain (PYD) at its N-terminus. Expression of the IFI16 protein is detectable in epithelial cells, fibroblasts, endothelial cells, macrophages, T cells, and cells of hematopoietic origin [25–28] in the nucleus (within the nucleus, both in the nucleolus and nucleoplasm), cytoplasm, or both [17, 29–31]. This unique protein architecture places IFI16 in a position to fulfill diverse roles within the immune system. IFI16-ASC inflammasomes are created when the IFI16 protein interacts with the ASC via the PYD. The protein’s N-terminus has a PYD domain, which suggests that it participates in the apoptotic pathway by controlling the activity of certain transcription factors that are linked to cell death in the nucleus [32].

The primary function of IFI16 lies in its role as a DNA sensor, particularly in the context of sensing cytosolic DNA [33]. The HIN domains of IFI16 exhibit an ability to specifically recognize double-stranded DNA, single-stranded DNA, or damaged nuclear DNA, allowing it to detect viral DNA, damaged host DNA, or even self-DNA released during cellular stress [34, 35]. This recognition mechanism positions IFI16 as a sentinel of potential threats to cellular integrity.

Upon binding to DNA, IFI16 undergoes conformational changes that facilitate its oligomerization. These assembled IFI16 complexes subsequently interact with the adaptor protein ASC, leading to the formation of an inflammasome complex [36]. This, in turn, triggers a cascade of immune responses, including the activation of caspase-1 and the release of pro-inflammatory cytokines like interleukin-1β (IL-1β) and interleukin-18 (IL-18) [37]. The activation of these cytokines propels the initiation of inflammation, further amplifying the immune response.

Furthermore, IFI16’s intricate involvement in antiviral defense mechanisms is noteworthy. During viral infections, IFI16 recognizes viral DNA, subsequently inducing the expression of type I interferons (IFNs) [38]. These IFNs play a critical role in alerting neighboring cells to the viral threat and triggering an antiviral state [39]. IFI16’s ability to modulate viral infection responses demonstrates its dynamic regulatory role in host defense mechanisms. Notably, IFI16’s functions extend beyond its role as a DNA sensor. Inflammatory stimuli, such as pathogen-associated molecular patterns (PAMPs) and danger-associated molecular patterns (DAMPs), can also activate IFI16-mediated signaling pathways [40, 41]. IFI16 senses PAMPs (human immunodeficiency virus type 1, Kaposi’s sarcoma-associated herpesvirus, vaccinia virus, human papillomavirus, and Ebola virus) through sensing double-stranded DNA, single-stranded DNA, and mRNA [1, 36, 42–44]. IFI16 is believed to have at least a partial role in responding to DAMPs that mediate the induction of interferon-β, IL-1β, and IL-18 [1, 32]. In addition, the presence of the downstream effectors CASP1 and HMGB-1 (a kind of DAMPs) in the inflamed mucosa indicates functional activation of major IFI16-mediated inflammasomes in the active inflammatory bowel disease colon [45]. However, the underlying mechanisms remain poorly understood. This versatile responsiveness positions IFI16 as a mediator between DNA damage, viral infections, and the initiation of inflammatory processes.

**Fig. 1:**
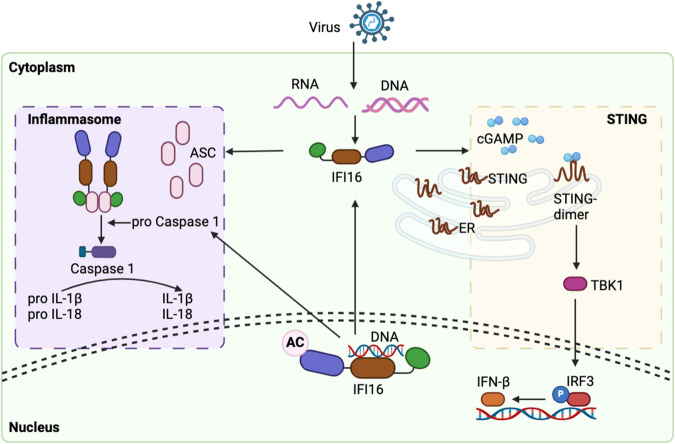
Structure and mechanism of IFI16.

In summary, as shown in Fig. 1, IFI16’s multi-domain architecture and unique recognition mechanisms enable it to serve as a crucial orchestrator of immune and inflammatory responses. Its ability to sense DNA, particularly viral DNA, and its engagement in inflammasome assembly highlight its role as a sentinel of cellular stress and danger. The intricate interplay between IFI16, DNA sensing, viral infections, and inflammatory pathways underscores its significance in maintaining immune homeostasis and shaping the host’s response to various threats.

## Cell death pathways and panoptosis

Cell death, a fundamental biological process, can occur through various distinct pathways, each contributing to diverse physiological and pathological contexts. Three primary cell death pathways are widely recognized: pyroptosis, apoptosis, and necroptosis [46].

Pyroptosis is an inflammatory form of cell death that plays a pivotal role in host defense against infections. It is characterized by cell swelling, plasma membrane rupture, and the release of pro-inflammatory cytokines [47]. Activation of pyroptosis typically involves the assembly of inflammasomes, protein complexes that include caspase-1 [48]. Inflammasomes are often triggered by the recognition of PAMPs or DAMPs, leading to rapid and highly regulated cell lysis [47].

Apoptosis, also known as programmed cell death, is a controlled and orderly process critical for tissue homeostasis and development. It is characterized by cell shrinkage, chromatin condensation, and membrane blebbing [49]. Apoptosis can be initiated through extrinsic pathways, triggered by death ligands binding to their respective death receptors, or intrinsic pathways, involving mitochondrial dysfunction and the release of apoptogenic factors [50]. Activation of caspases, proteases that orchestrate apoptotic events, leads to DNA fragmentation and ultimately the phagocytic clearance of apoptotic cells without inducing inflammation [51].

Necroptosis is a regulated form of necrotic cell death that is distinct from uncontrolled necrosis. It is initiated by death receptor signaling and involves the sequential activation of receptor-interacting serine/threonine-protein kinases (RIPKs), resulting in the formation of a necrosome complex [52]. Unlike apoptosis, necroptosis is characterized by cellular swelling, plasma membrane rupture, and the release of DAMP, contributing to inflammation and immune responses [53].

Recent research has unveiled the concept of PANoptosis, which highlights the interconnectedness and crosstalk between different cell death pathways. PANoptosis refers to the simultaneous activation of pyroptosis, apoptosis, and necroptosis, orchestrated by specific signaling molecules and molecular switches [11]. This concept underscores the dynamic nature of cell death regulation, where different pathways are activated in coordination to amplify immune responses and adapt to various stressors [54]. The intricate interplay between these cell death pathways is essential for maintaining tissue integrity, immune surveillance, and host defense [55]. The concept of PANoptosis underscores the need to consider the convergence of these pathways in understanding cellular fate decisions during infection, inflammation, and tissue damage. Investigating the regulatory mechanisms behind PANoptosis holds great promise for uncovering novel therapeutic strategies in diseases where dysregulated cell death contributes to pathogenesis.

## Mechanisms of IFI16 regulation of panoptosis

### Associations of IFI16 with different cell death pathways and PANoptosis regulation

IFI16’s versatile interactions with various cell death pathways underscore its potential role in orchestrating PANoptosis. IFI16 has been implicated in pyroptosis due to its involvement in inflammasome assembly and subsequent release of pro-inflammatory cytokines [56]. IFI16’s role in apoptosis is suggested by its modulation of apoptotic signaling pathways and potential influence on caspase activation [23, 31]. Furthermore, IFI16 can also induce cell death in the form of apoptosis and pyroptosis at the same time [3]. In addition, IFI16, MLKL, and RIPK1 have high expression in STAT3β overexpression cells to enhance necroptosis [57].

Based on these, the intricate web of IFI16’s functions in PANoptosis regulation may involve the integration of multiple signaling pathways. For instance, IFI16’s DNA sensing capability can initiate cascades of immune and inflammatory responses, influencing cell death decisions [58]. Additionally, IFI16’s interactions with adaptors and signaling molecules within inflammasomes [59], apoptotic complexes [60], and cell necroptosis [57] may collectively contribute to PANoptosis outcomes.

In summary, the interferon-inducible protein IFI16 has been implicated in the process of PANoptosis. PANoptosis plays a significant role in the pathophysiology of various diseases, including heart diseases, by orchestrating the interplay between these forms of cell death [61, 62]. Moreover, IFI16 is a key component in the IFI16-STING pathway, which is essential for initiating and regulating immune responses to DNA and RNA viruses [1, 3]. This pathway has been shown to induce the IFI16-dependent programmed cell death [63]. Interferon regulatory factor 3 (IRF3) is integral in the IFI16-STING pathway, where it acts downstream of STING to regulate the transcription of type I interferons and other genes involved in the antiviral response. IRF3, as a transcription factor, is also a powerful regulator of cardiometabolic diseases, responding to cardiometabolic stress by influencing various aspects of these diseases, including metabolic disorder, vascular injury, and cardiac hypertrophy [64]. This intricate connection suggests that targeting the IFI16-STING-IRF3 pathway and its associated PANoptosis mechanisms could offer novel therapeutic strategies for managing cardiometabolic diseases that have an inflammatory component, such as atherosclerosis. Thus, IFI16’s associations with different cell death pathways and its intricate role in PANoptosis regulation suggest a potential nexus for understanding heart disease pathogenesis. Deciphering the molecular intricacies of how IFI16 influences diverse cell death pathways and contributes to heart outcomes holds promise for unveiling new therapeutic strategies that target the intertwined processes of inflammation and cell death.

### Revealing the complex crosstalk: IFI16’s impact on inflammation, endothelial function, and PANoptosis

IFI16’s potential influence on multiple facets of heart disease emphasizes the need to explore its interconnected effects. By engaging with both inflammation [65] and endothelial function [66], IFI16 may mediate an inflammatory feedback loop, which could further fuel inflammatory responses to release more IL-1β and IL-18 [17]. IFI16’s involvement in multiple PANoptosis pathways potentially amplifies this loop, complicating the pathogenic process. Unraveling IFI16’s impact on PANoptosis regulation in heart diseases requires dissecting the molecular interactions underlying its functions. Investigating the crosstalk between IFI16, inflammation, and vascular endothelial function may provide insights into the intricate interplay of these components and their implications for disease progression [41, 67].

In conclusion, the role of PANoptosis regulation, orchestrated by IFI16, may unveil a new layer of complexity in heart diseases. IFI16’s potential to modulate multiple cell death pathways [4, 23], interact with inflammation [41], and influence vascular health [68] underlines its significance in shaping heart disease outcomes. A comprehensive understanding of IFI16-mediated PANoptosis may pave the way for novel therapeutic strategies targeting the intricate interplay of inflammation and cell death in heart diseases.

## Panoptosome in heart diseases

**Fig. 2:**
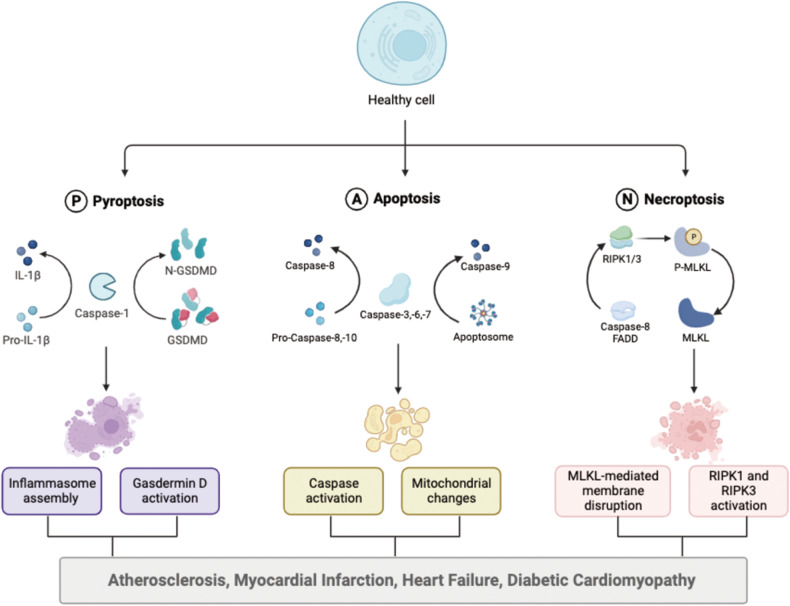
Mechanisms of PANoptosis in heart disease.

Heart diseases encompass a spectrum of conditions affecting the heart and blood vessels. In 2020, the crude prevalence of heart diseases is 607.64 million cases and 19.05 million deaths are estimated globally, attributed to various risk factors such as hypertension, dyslipidemia, smoking, and diabetes [69]. Pathophysiologically, heart diseases often involve chronic inflammation [70], endothelial dysfunction [71], oxidative stress [72], and cell death [73] contributing to disease progression. Mechanisms of PANoptosis in heart disease are shown in Fig. 2.

### Atherosclerosis

Atherosclerosis is a disorder that causes plaque to accumulate in the arteries and is a significant risk factor for heart problems. An essential part of the development of atherosclerotic plaque is the involvement of inflammatory processes in the arterial wall [74] (details in Table 1).

**Table 1:** Relationship between atherosclerosis and different cell death types.

Cell death type	Key findings	Reference
pyroptosis	This study highlights the role of IFN regulatory factor 1 (IRF-1) in mediating macrophage pyroptosis caused by oxidized low-density lipoprotein, a key factor in atherosclerosis. It suggests that IRF-1 induced pyroptosis contributes significantly to the pathogenesis of atherosclerosis and acute coronary syndrome.	[75]
	The paper discusses how pyroptosis, particularly through the activation of the NLRP3 inflammasome, is closely associated with cardiovascular diseases, including atherosclerosis. It suggests that targeting pyroptosis could be a promising therapeutic strategy for these conditions.	[76]
	This research indicates that nicotine exacerbates atherosclerosis through the induction of pyroptosis in endothelial cells, mediated by reactive oxygen species and the NLRP3 inflammasome. It underscores the pro-atherosclerotic effects of nicotine via pyroptosis pathways.	[77]
	While this paper broadly covers the mechanisms of pyroptosis and its implications in various diseases, including inflammatory diseases and cancer, it provides insights that are relevant to understanding pyroptosis in the context of atherosclerosis.	[78]
	It specifically focuses on GSDME-mediated pyroptosis and its role in exacerbating atherosclerosis. It demonstrates that inhibiting GSDME can reduce atherosclerotic lesion area and inflammation, suggesting a direct link between pyroptosis and atherosclerosis progression.	[80]
	It discusses the role of oxidative stress in atherosclerosis, which is relevant to understanding the oxidative stress-related pathways of pyroptosis in atherosclerotic processes.	[81]
	This paper explores the role of PCSK9 in inflammation and atherosclerosis. While it may not directly address pyroptosis, the inflammatory pathways discussed could intersect with pyroptotic processes in atherosclerosis.	[82]
	This review systematically illustrates how ROS triggers various forms of endothelial cell death, including pyroptosis. It provides valuable insights into the mechanisms by which pyroptosis may contribute to endothelial dysfunction and atherosclerosis.	[83]
apoptosis	It focuses on the role of IRGM/Irgm1 in macrophage apoptosis and its impact on atherosclerotic plaque stability. It suggests that IRGM levels in patients could predict plaque rupture and that Irgm1 deficiency in mice leads to reduced necrotic plaque cores, indicating increased plaque stability.	[84]
	This review discusses the role of foam cells in atherosclerosis, highlighting their origins and molecular mechanisms. It notes that foam cells undergo various programmed cell death pathways, including apoptosis, contributing to the necrotic cores of atherosclerotic plaques.	[85]
	This paper explores the role of TET2 in vascular smooth muscle cell apoptosis and its implications for transplant vasculopathy. It suggests that TET2 expression is negatively regulated in human coronary allograft vasculopathy and that TET2 depletion results in increased apoptosis and medial thinning.	[86]
	This article discusses the importance of imaging apoptosis in atherosclerotic plaques for detecting plaque instability. It highlights that apoptotic macrophage death enlarges the plaque necrotic core and affects the stability of the plaque.	[87]
	This study emphasizes the crucial role of macrophage apoptosis and efferocytosis in atherosclerosis progression and plaque stability. It discusses how environmental stressors and endoplasmic reticulum stress can initiate apoptosis in macrophages.	[88]
	This review focuses on efferocytosis, the process of clearing apoptotic cells, and its importance in maintaining tissue homeostasis. It discusses how defective efferocytosis contributes to chronic inflammatory diseases, including atherosclerosis.	[89]
necroptosis	It shows that necroptotic cell death, regulated by RIP3 and MLKL kinases, is activated in human-advanced atherosclerotic plaques. The study suggests that targeting necroptosis can be a therapeutic and diagnostic approach, as evidenced by the use of a radiotracer developed with the necroptosis inhibitor necrostatin-1, which localizes specifically to atherosclerotic plaques in mice and reduces lesion size and markers of plaque instability.	[90]
	This paper discusses the role of necroptosis, a pro-inflammatory process of programmed cell necrosis, in atherosclerosis. It emphasizes that necroptosis may promote the growth of the necrotic core in atherosclerotic plaques and could be a novel target for molecular imaging and translational therapeutic interventions.	[91]
	This review explores the role of necroptosis in vascular endothelial cells, vascular smooth muscle cells, and macrophages in atherosclerotic plaques. It highlights that necroptosis, a caspase-independent programmed cell necrosis, occurs in advanced atherosclerotic plaques and plays an important pathophysiological role in accelerating the disease process.	[92]

Pro-inflammatory cytokines including IL-18, IL-1β, and monocyte chemoattractant protein-1 are released during the process of pyroptosis [75]. These cytokines accelerate the development and instability of atherosclerotic plaques [76, 77]. Multiprotein complexes called inflammasomes are essential for the start of pyroptosis. Additionally, they are connected to the inflammatory process in atherosclerotic plaques [78]. Plaque inflammation is made worse by the activation of inflammasomes in macrophages and endothelial cells inside the plaque, which results in the production of pro-inflammatory cytokines. One important effector molecule in pyroptosis is gasdermin D (GSDMD). Furthermore, new information indicates that the GSDMD and GSDME proteins—which create holes in the membranes of mitochondria and plasma—are essential to the pyroptosis process [79]. In addition to promoting the production of pro-inflammatory cytokines, these proteins cause mitochondrial malfunction and heighten the pro-inflammatory response in atherosclerosis. Gaining insight into these molecular pathways paves the way for therapeutic targeting of pyroptosis to control inflammation and may be slow down the course of AS [80]. Oxidized low-density lipoprotein (LDL) cholesterol may leak out of atherosclerotic plaques when macrophages undergo pyroptosis [81]. This released cholesterol has the potential to worsen lipid buildup in the artery wall, causing oxidative stress [82] and extending the cycle of inflammation and plaque formation [83]. Endothelial cell pyroptosis also weakens the vascular wall’s structural integrity and increases the risk of thrombosis and plaque rupture [84].

Apoptosis may facilitate macrophage removal from atherosclerotic plaques. As a result of their ability to consume fats and produce foam cells, macrophages are essential to the development of atherosclerosis [85]. These foam cells may undergo apoptosis and contribute to the necrotic cores of atherosclerotic plaques [86]. In atherosclerotic lesions, vascular smooth muscle cells also go through apoptosis. This may weaken a plaque’s fibrous cap and increase the likelihood of a rupture [87]. Thrombotic events, such as heart attacks or strokes, can be brought on by ruptured plaques. Chemokines and cytokines, signaling molecules released by apoptotic cells, draw immune cells to the site of cell death. The infiltration of immune cells inside atherosclerotic plaques has the potential to intensify inflammation, hence exacerbating plaque instability [88]. Phagocytes remove apoptotic cells by a non-inflammatory mechanism called efferocytosis. The efferocytosis of apoptotic cells by macrophages contributes to the stability of the plaque in atherosclerosis by limiting the buildup of apoptotic debris [89], which can exacerbate inflammation [90].

Atherosclerosis development has been linked to necroptosis, a kind of necrosis that is regulated. Necroptosis is far more pro-inflammatory and caspase-independent than apoptosis. Necroptosis impacts smooth muscle cells, macrophages, and vascular endothelial cells inside the environment of atherosclerotic plaques, which adds to the instability of the plaque. The phosphorylation of mixed lineage kinase domain-like protein and RIPK3 is one of the biochemical processes underlying necroptosis mixed lineage kinase domain-like pseudokinase (MLKL). It is well known that atherogenic lipoproteins, especially LDL can cause RIPK3 and MLKL to be phosphorylated transcriptionally. These are critical stages in the necroptotic process [91]. Moreover, the development of the necrotic core and the advancement of plaque have been connected to the existence of necroptosis in human atherosclerotic tissues [92]. Macrophages are a major target for therapeutic therapies meant to lessen the burden of atherosclerosis since they are essential to inflammation and necroptosis within plaques [91]. Macrophages infiltrate the vascular intima, activate endothelial cells, and attract monocytes to the vessel wall. These monocytes differentiate into macrophages, ingesting modified lipoprotein to form foam cells [93]. These foam cells die through apoptosis and become necrotic, leading to plaque destabilization and rupture [94]. Macrophages further fuel lesion inflammation through cytokine secretion and proteolytic activity, leading to atherothrombosis and ischemic events [95]. These results demonstrate the potential of necroptosis as a novel target for therapeutic intervention as well as a biomarker for the advancement of atherosclerosis [96].

PANoptosis promotes crosstalk and co-regulation among various cell death pathways, both of which are important in the course of atherosclerotic disease [10]. The AIM2 inflammasome, for example, has been demonstrated to modulate innate immune sensors and induce inflammatory signaling, therefore contributing to the inflammatory milieu of atherosclerosis [97]. Furthermore, the combination of molecules from the pyroptotic, apoptotic, and necroptotic pathways results in the formation of the PANoptosome, which is triggered by a variety of pathogenic events, including those implicated in atherosclerosis [98]. This intricate interaction of cell death pathways in PANoptosis highlights the multiple character of atherosclerosis, which is both a fat-storage disease and an inflammatory condition [99].

### Myocardial infarction (MI)

MI is a disorder marked by an abrupt cessation of blood supply to the heart muscle, leading to cardiomyocyte death [100] (details in Table 2).

**Table 2:** Relationship between myocardial infarction (MI) and different cell death types.

Cell death type	Key findings	Reference
pyroptosis	It discusses the role of pyroptosis in MI, highlighting how this process contributes to inflammation and cell death in the heart. The paper also explores potential therapeutic strategies to regulate pyroptosis, which could be beneficial in treating MI by reducing inflammation and preserving cardiac function.	[78]
	It details how pyroptosis contributes to the pathophysiology of MI by promoting inflammation, exacerbating tissue damage, and influencing the remodeling of cardiac tissue. The paper also discusses the molecular pathways involved in pyroptosis, such as the activation of caspases and gasdermin D, and how these pathways are potential targets for therapeutic intervention in MI.	[100]
	This study emphasizes the impact of pyroptosis on cardiac cell death, inflammation, and subsequent heart failure following a MI. The paper also examines the signaling pathways that lead to pyroptosis in cardiac cells and discusses how understanding these pathways could lead to new treatments for MI that focus on reducing cell death and inflammation.	[103]
	This paper explains how pyroptosis contributes to the progression of MI by inducing inflammatory responses and cell death in the heart. It also explores various molecular mechanisms and signaling pathways involved in pyroptosis, such as inflammasomes and gasdermin proteins, and their potential as therapeutic targets in MI.	[104]
	It highlights the role of pyroptosis in exacerbating heart damage during MI by promoting inflammation and cell death. The paper also reviews the molecular pathways leading to pyroptosis and suggests that targeting these pathways could be a novel approach to treat MI by reducing inflammatory responses and preserving heart tissue.	[105]
apoptosis	It discusses how apoptosis contributes to the loss of cardiac myocytes during and after a MI, leading to impaired cardiac function and heart failure. The paper also explores the molecular mechanisms underlying apoptosis in MI, including the activation of caspases and the role of mitochondrial dysfunction.	[106]
	Bennett’s research highlights the significance of apoptosis in the pathogenesis of MI, detailing how apoptotic cell death of cardiac cells contributes to the remodeling of the heart and the progression of heart failure. The paper also reviews the potential therapeutic strategies to modulate apoptosis in the context of MI.	[107]
	This article examines the occurrence and implications of apoptosis in myocardial ischemia and infarction. It emphasizes the dual role of apoptosis in MI, both as a mechanism of cell loss and as a potential protective response to limit necrosis and inflammation. The paper also discusses the triggers and regulators of apoptosis in the ischemic myocardium, including ischemia-reperfusion injury and inflammatory responses.	[108]
	This study investigates the role of the MEK1-ERK2 signaling pathway in protecting the myocardium from ischemic injury. It demonstrates that activation of this pathway can reduce apoptosis in cardiac cells during MI, thereby preserving cardiac function and reducing infarct size. The paper suggests that modulation of this pathway could be a therapeutic strategy to protect the heart from ischemia-induced apoptosis.	[110]
	Saraste et al. provides evidence of apoptotic cell death in the cardiac tissue of patients who suffered from MI, highlighting its contribution to myocardial damage and the subsequent weakening of cardiac function. The study underscores the importance of apoptosis as a therapeutic target in the treatment of MI.	[111]
	This research explores the therapeutic potential of inhibiting programmed cell death pathways, including apoptosis, in improving cardiac function after MI. The study demonstrates that pharmacological inhibition of apoptosis, along with necroptosis and ferroptosis, can significantly improve left ventricular function in rats post-MI. It suggests that targeting these cell death pathways could be an effective strategy in the treatment of MI.	[112]
	This study introduces the anti-apoptotic long non-coding RNA Sarrah, which is regulated by aging and plays a role in recovery from acute MI. The paper demonstrates that Sarrah can reduce apoptosis in cardiac cells, thereby enhancing cardiac repair and function after MI. It highlights the potential of targeting Sarrah as a novel therapeutic approach in the treatment of MI, especially in the aging population.	[113]
necroptosis	It highlights how necroptosis contributes to cardiac cell death and exacerbates heart damage during MI. The paper also explores the molecular mechanisms of necroptosis, including the involvement of RIPK1, RIPK3, and MLKL, and suggests that targeting necroptosis could be a novel therapeutic strategy for MI.	[114]
	This article reviews the pathophysiology of necroptosis and the therapeutic implications of its inhibition, particularly in the context of MI. It discusses the potential of necrostatin-1, an inhibitor of necroptosis, in reducing cardiac cell death and improving outcomes in MI. The paper also delves into the signaling pathways involved in necroptosis and how their modulation could offer new avenues for MI treatment.	[115]
	It reviews various pharmaceutical therapies targeting necroptosis and their potential to mitigate myocardial damage. The paper emphasizes the importance of understanding necroptosis in the context of MI and explores different drugs that can inhibit this cell death pathway to improve cardiac outcomes.	[116]
	It details the molecular mechanisms underlying necroptosis in the heart and how this form of cell death contributes to myocardial damage and heart failure. The paper also discusses potential therapeutic strategies to inhibit necroptosis, which could be beneficial in treating MI by reducing cardiac cell death and inflammation.	[117]
	It highlights how necroptosis contributes to the pathogenesis of MI by inducing cell death and inflammatory responses in the heart. The paper also reviews the signaling pathways involved in necroptosis and discusses the potential of targeting these pathways as a therapeutic approach in MI.	[118]

There is growing recognition that pyroptosis plays a major role in MI pathophysiology. The cleavage of GSDMD and subsequent activation of caspase-1 are the hallmarks of this type of programmed cell death [101, 102]. This process creates holes in the cell membrane and releases inflammatory cytokines such as IL-1β and IL-18. Then, this inflammatory cascade is crucial because it exacerbates inflammation and may have an effect on plaque stability, both of which increase the risk of myocardial injury in MI [80, 103]. The significance of the inflammasome in this process has been brought to light by recent research, especially that of the NLR family pyrin domain-containing 3 (NLRP3) inflammasome, which recognizes stress signals and cellular damage and activates caspase-1. Not only does the cleavage of GSDMD by caspase-1 result in cell death, but it also stimulates the release of IL-1β and IL-18, which can draw more immune cells to the site of damage, increasing inflammation and hastening the course of MI [104, 105]. Moreover, pyroptosis has been implicated in various stages of cardiovascular diseases, including atherosclerosis, which is a key underlying condition that predisposes individuals to MI. The involvement of pyroptosis in the destabilization of atherosclerotic plaques can lead to thrombus formation and subsequent MI. The understanding of these molecular mechanisms provides insight into new potential therapeutic targets, such as inhibitors of caspases or blockers of GSDMD pores, which could mitigate the inflammatory response and reduce myocardial damage [105, 106]. Targeting this cell death process may be a unique strategy for treating or avoiding the negative effects associated with MI, according to the continuing study on pyroptosis and its role in the disease. Therefore, pyroptosis regulation presents a viable therapeutic intervention method to enhance the prognosis of patients with MI and other associated cardiovascular disorders [80, 106]. To summarize, the activation of inflammatory caspases and GSDMD, which results in cell death and the production of cytokines that worsen cardiac damage, is how pyroptosis contributes to MI. This knowledge creates new opportunities for treatment approaches meant to reduce inflammation and preserve heart tissue after MI. More specifically, NLRP3, IL-18, and IL-1β inhibition can prevent caspase-1 activation. Thus, the pyroptosis of cardiomyocytes was inhibited to protect the death of cardiomyocytes.

When there is a MI, the myocardium’s blood supply is cut off, causing ischemia and the consequent death of cardiomyocytes. Apoptosis plays a role in the loss of these cells in the surrounding and ischemic core regions, influencing the infarct’s size and severity [107]. A balance between pro- and anti-apoptotic signals inside the heart cells is one of the molecular causes of apoptosis in MI. Caspases are essential for the execution stage of apoptosis, and their activation is one of the pro-apoptotic signals. Apoptosis is characterized by caspase activation, which causes DNA destruction and the breakage of structural proteins in the cytoskeleton. The Bcl-2 protein family, which consists of apoptosis promoters and inhibitors, controls these processes [108]. Moreover, myocardial apoptosis is made worse by ischemia-reperfusion damage, a situation that happens when blood flow to the tissue resumes after an ischemic phase [109]. This is because reactive oxygen species and calcium overload are produced, which trigger apoptosis signaling pathways [110]. Conversely, it has been demonstrated that anti-apoptotic pathways, such as the MEK1-ERK1/2 signaling pathway, shield the heart against ischemia damage. Through the prevention of DNA breakage and preservation of heart function during ischemia-reperfusion damage, activation of this pathway can limit apoptosis [111]. Furthermore, it is becoming more widely acknowledged that non-coding RNAs and microRNAs have a role in controlling apoptosis. For example, it has been discovered that microRNA-155 stimulates apoptosis in MI by focusing on certain RNA-binding proteins that are crucial for cell survival [112]. Apoptosis has drawn attention as a potential treatment target for MI in recent years. One possible tactic to lessen the loss of cardiomyocytes and enhance cardiac function following MI is to pharmacologically suppress the pathways involved in programmed cell death [113]. Another possibility for therapeutic targets is the investigation of anti-apoptotic long non-coding RNAs, which may provide protection against cardiomyocyte death [114]. In conclusion, a complex interaction of molecular signals causes apoptosis to have a substantial influence on MI. The degree of cell death and the final result after a MI depend on the balance between the pro- and anti-apoptotic pathways. Knowing these pathways offers important information about possible treatment approaches that can enhance recuperation and lessen the morbidity related to MI.

Necroptosis has been found to have a crucial role in the pathophysiology of MI, indicating that inhibiting it may be a useful therapeutic target. In animal models, pharmacological inhibitors that selectively target RIPK1, including necrostatin-1 have been demonstrated to decrease infarct size and myocardial cell death [115, 116]. Since RIP1 is essential to the necroptosis pathway, blocking it has been shown to preserve the structural integrity of the heart and prevent the reactive fibrotic process from occurring after myocardial ischemia/reperfusion [117]. Furthermore, it has been suggested to employ pharmacological inhibitors that specifically target MLKL and RIPK3, two more elements of the necroptosis signaling cascade. By obstructing the necroptosis pathway, these therapeutic medicines may have positive effects in clinical diseases including MI and HF by reducing inflammation and preventing cell death [118]. Necroptosis has a part in cardiovascular disorders that go beyond MI. It is thought to have a major role in the pathogenesis of diseases, including cardiac remodeling and vascular atherosclerosis. As a result, focusing on necroptosis signaling pathways may have wide-ranging therapeutic effects when treating different cardiovascular illnesses [119]. To sum up, necroptosis is a possible therapeutic target for MI that might lead to better clinical results. It may be possible to lessen overall myocardial damage and improve myocardial recovery after a MI by inhibiting important molecules in the necroptosis process. The goal of this field’s current research is to create new therapies for MI and other cardiovascular conditions where necroptosis is a factor. These results highlight how crucial it is to comprehend necroptosis and how it contributes to heart disease in order to guide the creation of tailored treatments that may help people with cardiovascular diseases.

The link between PANoptosis and MI is gaining attention, revealing the intricate interaction of cell death pathways in heart damage. For example, after MI, activation of these cell death pathways might increase myocardial damage, leading to poorer outcomes [120]. The PANoptosome, a molecular complex crucial to PANoptosis, plays an important role in MI, where its activation leads to the inflammatory response and myocardial damage [121]. Furthermore, evidence suggests that targeting PANoptosis components such as inflammasomes might provide new treatment methods for MI, potentially lowering myocardial damage and increasing cardiac function post-infarction [122]. This demonstrates the promise of PANoptosis-targeted treatments in MI care, providing a unique method for mitigating the effects of acute myocardial damage [123].

### Heart failure (HF)

The prevalence of HF ranges between 1 and 3% in the general adult population, a disease in which the heart cannot adequately pump blood [124]. Recent research emphasizes the significance of early detection and targeted treatment techniques. For example, a study emphasizes the significance of sodium-glucose cotransporter 2 inhibitors in improving outcomes for patients with HF and low ejection fraction, independent of diabetes status. These findings highlight the changing environment of HF care, with an emphasis on customized therapy and innovative therapeutic methods [125] (details in Table 3).

**Table 3:** Relationship between heart failure (HF) and different cell death types.

Cell death type	Key findings	Reference
pyroptosis	This paper discusses the role of pyroptosis in cardiovascular diseases, including HF. Pyroptosis, characterized by caspase-1-dependent formation of plasma membrane pores, leads to the release of pro-inflammatory cytokines and cell lysis. The activation of the NLRP3 inflammasome, linked to cardiovascular risk factors, is closely associated with pyroptosis. The study suggests that targeting NLRP3 inflammasome and pyroptosis could be promising for treating cardiovascular diseases like HF.	[76]
	This review highlights the involvement of pyroptosis in various cardiovascular diseases, including HF. Pyroptosis, a form of programmed necrosis, is driven by caspase-1 and caspase-4/5/11 signaling pathways. The paper suggests that pyroptosis signaling pathways may be potential therapeutic targets in cardiovascular diseases.	[100]
	This review summarizes the molecular mechanisms, regulation, and cellular effects of pyroptosis in cardiovascular diseases. It discusses the role of pyroptosis in HF, emphasizing the development of therapeutic approaches based on the regulation of pyroptosis.	[103]
	The study focuses on the role of pyroptosis in cardiac remodeling, a fundamental mechanism of HF. It explains how pyroptosis contributes to cardiac fibrosis, hypertrophy, myocardial dysfunction, and excessive inflammation, leading to cardiac remodeling in HF. The paper discusses the potential of targeting pyroptosis to improve cardiac remodeling in HF.	[125]
apoptosis	This paper outlines how HF, resulting from cardiac injury or overload, leads to structural changes in the heart, including apoptosis in cardiomyocytes. This apoptosis contributes to systolic dysfunction and further heart deterioration	[126]
	This paper discusses the role of apoptosis in the progression of HF. It emphasizes that apoptosis, or programmed cell death, of cardiac muscle cells, is a key process in cardiac remodeling, which precedes HF. The paper reviews the apoptotic pathways in cardiac muscle and suggests that understanding these pathways is crucial for developing future treatments for HF.	[127]
	In this article, apoptosis is identified as a critical factor in HF, with the process being triggered in adult cardiac cells due to factors like ventricular dilatation and neurohormonal activation leading to cell loss and a decline in ventricular function.	[128]
	This study explores the role of RUNX1 in HF progression. It finds that inhibition of RUNX1 leads to a decrease in cardiac enlargement and myocardial fibrosis, as well as a reduction in the rate of apoptotic cell death in myocardial tissues. The study suggests that RUNX1, by activating TGF-β/Smads signaling, could be a new therapeutic target against HF.	[129]
	Apoptosis in HF is predominantly driven by factors like oxidants, excessive nitric oxide, angiotensin II, and catecholamines, which trigger caspase activation leading to cardiomyocyte death, a key aspect in the progression of HF.	[130]
necroptosis	The study found that RIPK1-independent necroptosis contributes to the pathogenesis of DIC, and treatment with rapamycin significantly improves heart function in DIC by reducing MLKL activity and preventing necroptosis.	[131]
	This paper discusses the mechanisms of cell death, including apoptosis, necrosis, and autophagy. It highlights that necrosis, once considered a passive form of cell death, is now recognized as a programmed process with significant biological consequences, including the induction of an inflammatory response. This understanding is crucial in the context of HF, where cell death mechanisms play a pivotal role.	[132]
	This study focuses on the role of Serinc2 in sepsis-induced cardiomyopathy (SIC), a condition characterized by decreased ventricular contractility. The findings suggest that Serinc2 deficiency exacerbates myocardial inflammation, necroptosis, apoptosis, and myocardial damage in SIC. The study indicates that targeting Serinc2 could be a potential therapeutic approach for HF associated with sepsis.	[133]

In HF pathophysiology, pyroptosis is a major factor in the inability of the heart to pump blood efficiently enough to fulfill the body’s demands. GSDMD and GSDME proteins produce membrane holes, which cause cell lysis and swelling as well as the release of pro-inflammatory cytokines such as IL-1β and IL-18 [103, 104]. These cytokines have been linked to the development of many cardiac diseases, such as cardiac fibrosis and myocardial hypertrophy, and they also contribute to the inflammatory milieu associated with HF. Often occurring before HF, myocardial hypertrophy is a compensatory reaction to cardiac stress that is made worse by the inflammatory response brought on by pyroptosis. Particularly, IL-1β and IL-18 are known to draw in more immune cells, which exacerbates inflammation and aids in pathological remodeling [106]. Significant elements of cardiac remodeling, a process linked to the decline in cardiac function in HF, include cardiac fibrosis and hypertrophy. In addition to killing cardiomyocytes, pyroptosis also induces excessive inflammation, which results in the deposition of fibrotic tissue and myocardial hypertrophy, which all contribute to this remodeling [126]. Mechanistically, two signaling pathways—one mediated by caspase-1 and the other by caspase-4/5/11—are the main drivers of pyroptosis. When these caspases are activated, GSDMD and GSDME cleave and go to the cell membrane to create holes, which causes cell death and the release of inflammatory substances [103]. In HF, focusing on the molecular processes of pyroptosis provides a treatment option. Myocardial structure and function may be preserved by reducing the inflammatory response by inhibiting pyroptosis pathway components like caspases and inflammasomes. This strategy may prevent heart remodeling and enhance clinical results for HF patients [77]. In conclusion, pyroptosis induces inflammation and affects cardiac remodeling, both of which contribute to HF. Modulating pyroptosis via interventions may be a unique approach to treating HF and may offer a technique to slow the course of this crippling illness.

Following cardiac damage, such as MI or elevated hemodynamic stress, the process of apoptosis in cardiomyocytes is characterized by cellular, structural, and neurohumoral alterations that contribute to the loss of cardiac function [127]. Apoptosis causes the death of cardiac myocytes in the context of HF, which is a crucial aspect of the illness’s development. The pathogenesis of HF is largely dependent on the weakening of the myocardial wall, dilatation of the heart chambers, and loss in contractile ability that arise from these cells dying through apoptosis [128]. Moreover, the hallmarks of HF- ventricular dilatation and neurohormonal stimulation- might cause apoptosis by upregulating transcription factors that support cell death [129]. The heart’s response to damage and stress is known as cardiac remodeling, and it involves structural alterations, myocardial cell enlargement, and an increase in apoptosis. The remodeling process is intimately associated with the advancement of HF, as the heart’s capacity to contract efficiently and sustain sufficient blood circulation is compromised by the death of cardiomyocytes [130]. According to recent studies, HF symptoms and cell preservation may benefit from caspase inhibitor-induced apoptosis inhibition, both in vivo and in vitro. These inhibitors may be able to stop the progression of HF by blocking the execution phase of apoptosis, which would improve patient outcomes [131]. Because of the intricate pathophysiology of HF, it is essential to have a thorough grasp of apoptosis and how it affects cardiac function. Finding new therapeutic targets within the apoptotic pathway might potentially improve HF management and therapy while also reducing the condition’s impact on patients’ quality of life.

Necroptosis is known to be a major factor in the development of HF, a disorder marked by the heart’s incapacity to pump blood effectively. RIPK1 and RIPK3 trigger this kind of cell death, which phosphorylates MLKL. Necroptosis is characterized by the loss of plasma membrane integrity brought on by MLKL activation [132]. The intricate signaling pathways involved in HF necroptosis regulation are part of the molecular processes. As an example, TAK1, a crucial protein that enhances cardiac cell survival, has been found to be a direct antagonist of necroptosis, contributing to myocardial remodeling and myocardial homeostasis control [119]. Additionally, new studies have shed light on the regulation of necroptosis in the heart and its significance for the etiology of diseases including MI and HF [133]. Necroptosis is involved in myocardial homeostasis maintenance as well as cardiac remodeling and ischemia-reperfusion injury. Therefore, pharmaceutical strategies that target necroptosis signaling pathways may be beneficial for treating cardiovascular illnesses, such as HF. Necroptosis has also been shown to exacerbate conditions such as sepsis-induced cardiomyopathy, underscoring the importance of this process of cell death in the progression of heart disease [134]. Ultimately, the distinct molecular pathways associated with necroptosis are connected to the ailment’s remodeling and degeneration. Understanding these pathways is crucial to developing specialized medicines that might impede the progression of HF and improve the prognosis of patients afflicted with this debilitating condition.

The relationship between PANoptosis and HF is an intricate one, highlighting the complex interplay of cell death pathways in cardiac dysfunction. RNA-binding proteins, which are vital in post-transcriptional gene regulation, have been linked to PANoptosis in HF, indicating a significant role in the disease’s progression. The PANoptosome is involved in various HF subtypes, characterized by different pathway activities and levels of PANoptosis genes [135]. This suggests that the dysregulation of PANoptosis could contribute to the development and progression of HF, offering new avenues for treatment and management of this complex condition.

### Diabetic cardiomyopathy (DCM)

DCM is a complicated syndrome defined by myocardial dysfunction in the absence of coronary artery disease and hypertension. It is associated with metabolic problems such as glucose toxicity and lipotoxicity as a result of insulin resistance. Capillary injury, cardiac fibrosis, and hypertrophy with mitochondrial dysfunction are all important characteristics. Increased oxidative stress and inflammation also contribute to the advancement of DCM. Current treatments are aimed at treating these metabolic anomalies, but new focused therapeutics are required [136] (details in Table 4).

**Table 4:** Relationship between diabetic cardiomyopathy (DCM) and different cell death types.

Cell death type	Key findings	Reference
pyroptosis	This study found that mitochondrial damage and activation of the cytosolic DNA sensor cGAS-STING pathway leads to cardiac pyroptosis and hypertrophy in DCM mice. It highlights the role of mitochondrial dysfunction and subsequent activation of inflammatory pathways in promoting pyroptosis in DCM.	[136]
	The research demonstrated that METTL14 suppresses pyroptosis and DCM by downregulating TINCR lncRNA. It suggests a molecular mechanism where METTL14 acts as a protective factor against pyroptosis in DCM through the regulation of lncRNA.	[137]
	This study showed that co-administration of hydrogen and metformin exerts cardioprotective effects by inhibiting pyroptosis and fibrosis in DCM. It indicates the therapeutic potential of combining hydrogen and metformin in reducing pyroptosis and improving cardiac function in DCM.	[138]
	The paper discusses the pyroptosis-related inflammasome pathway as a new therapeutic target for DCM. It emphasizes the significance of the inflammasome pathway in the pathogenesis of DCM and its potential as a therapeutic target.	[139]
	This research found that Cyclovirobuxine D ameliorates experimental DCM by inhibiting cardiomyocyte pyroptosis via NLRP3 both in vivo and in vitro. It suggests a novel molecular target for the clinical application of Cyclovirobuxine D in DCM.	[140]
	The study revealed that Pyrroloquinoline quinone ameliorates DCM by inhibiting the pyroptosis signaling pathway in C57BL/6 mice and AC16 cells. It highlights the protective effects of Pyrroloquinoline quinone against DCM through the inhibition of pyroptosis.	[141]
apoptosis	This study found that Circ-CDR1as is upregulated in DCM (DCM) and its knockdown can improve apoptosis caused by DCM. It activates the Hippo signaling pathway by inhibiting Mammalian sterile 20-like kinase 1 ubiquitination. Forkhead box group O3a (FOXO3), a transcriptional factor of CDR1as, activates the Hippo signaling pathway. The study also highlights the role of m6A methylation in DCM, particularly the ALKBH5-FOXO3-CDR1as/Hippo signaling pathway.	[142]
	The study investigates the effect of LCZ696 on DCM in rats. LCZ696 and valsartan were found to ameliorate DCM progression by inhibiting AGEs formation, pro-apoptotic markers, and NF-κB. They also restored elevated pro-inflammatory cytokines and ER stress parameters. The study concludes that LCZ696 has a strong protective effect against DCM by inhibiting myocardial inflammation, ER stress, and apoptosis.	[143]
	This study shows that Scutellarin (SCU) can improve type 2 DCM by regulating cardiomyocyte autophagy and apoptosis. SCU downregulated blood glucose, cholesterol, and triglyceride levels, improved myocardium morphology, and reduced myocardial apoptosis. It promoted autophagy-related factors and inhibited apoptosis-related factors, suggesting its potential in relieving T2DC.	[144]
	The study found that the soluble epoxide hydrolase (sEH) inhibitor AUDA attenuates cardiac remodeling and dysfunction in DCM by increasing autophagy and reducing apoptosis. This effect is related to the activation of the Nrf2 signaling pathway. AUDA upregulated Nrf2 expression and promoted its nuclear translocation, suggesting its therapeutic potential in DCM.	[145]
necroptosis	This study investigates the protective effect of hydrogen sulfide (H2S) on DCM, focusing on necroptosis. It found that H2S deficiency led to increased mitochondrial damage, reactive oxygen species accumulation, and activation of necroptosis and NLRP3 inflammasome. Conversely, supplementation with exogenous H2S improved DCM by alleviating necroptosis, suppressing NLRP3 inflammasome activation, and reducing oxidative stress.	[146]
	Sirtuin 3 (SIRT3) deficiency was found to worsen DCM by enhancing necroptosis and activating the NLRP3 inflammasome. The study underscores the importance of SIRT3 in regulating necroptosis and suggests that modulating SIRT3 activity could be a potential therapeutic approach for DCM.	[147]

Pyroptosis is a specific type of programmed cell death that has been the subject of growing amounts of recent study in the field of DCM. Numerous biological pathways and possible therapeutic targets in this field have been identified by studies. The cGAS-STING signaling pathway, which is active in diabetic hearts, has been the focus of one line of research. Targeting this route may help reduce ventricular dysfunction in DCM since it triggers NLRP3 inflammasome activation, cardiac pyroptosis, and persistent inflammation [137]. An additional investigation emphasized the significance of epigenetic control in pyroptosis, with a specific emphasis on METTL14-mediated m6A methylation of the TINCR lncRNA, which is pivotal in inhibiting pyroptosis and the advancement of DCM [138]. Furthermore, research on the therapeutic potential of hydrogen treatment for DCM has been conducted. It was discovered that inhaling hydrogen reduced pyroptosis and fibrosis, attenuating DCM and suggesting a new course of therapy [139]. Targeting the pyroptosis-related inflammasome pathway as a novel treatment approach has been highlighted by a study of the roles played by pyroptosis and the inflammasome in the development of DCM [140]. The effects of substances like pyrroloquinoline quinone (PQQ) and cyclovirobuxine D (CVB-D) on DCM have been the subject of several investigations [141, 142]. It has been demonstrated that CVB-D considerably reduces myocardial pathology in DCM by blocking cardiomyocyte pyroptosis through NLRP3 [141]. PQQ also enhanced DCM by blocking the NF-κB/NLRP3 inflammasome-mediated cell pyroptosis, indicating that it might be used as a dietary supplement to treat DCM [142].

Studies have demonstrated that DCM causes an upregulation of circular RNA cerebellar degeneration-related protein-1 antisense and that apoptosis brought on by DCM may be ameliorated by its suppression. This is accomplished by controlling the ubiquitination levels of Mammalian sterile 20-like kinase 1 and activating the Hippo signaling pathway [143]. Different studies revealed that the medication LCZ696 slows down the course of DCM by blocking apoptosis via the AGEs/NF-κB and PERK/CHOP signaling pathways, suggesting that it may be useful as a therapy [144]. Moreover, it has been determined that circHIPK3, a circular RNA that is downregulated in DCM, is essential for controlling apoptosis in cardiomyocytes. By suppressing the tumor suppressor gene PTEN, overexpression of circHIPK3 shields cardiomyocytes from high glucose-induced cell death [145]. Furthermore, it has been demonstrated that the soluble epoxide hydrolase inhibitor AUDA protects against DCM by promoting autophagy and lowering apoptosis, two processes that are connected to the Nrf2 signaling pathway’s activation [146]. All of these investigations show how intricately different molecular mechanisms interact to control apoptosis in DCM. They highlight the possibility of concentrating on these pathways to create DCM therapies that are successful in lowering apoptosis and enhancing heart function.

According to research, hydrogen sulfide reduces necroptosis, which protects against DCM. This is accomplished by preventing reactive oxygen species buildup and mitochondrial damage, which in turn lowers necroptosis and NLRP3 inflammasome activation [147]. Furthermore, the influence of Ca^2+^/calmodulin-dependent protein kinase II activation on DCM necroptosis was investigated. According to the findings, DCM causes an increase in CaMKII activation and necroptosis in a RIPK3-dependent way, which may provide new avenues for treatment [105]. Additionally, research on the function of Sirtuin 3 (SIRT3) in DCM showed that a SIRT3 deficit worsens the disease by increasing necroptosis and triggering the NLRP3 inflammasome. This implies that SIRT3 may be a target for molecular interventions in DCM prevention and therapy [148].

The connection between PANoptosis and DCM is a new field of study that highlights the complicated interaction of cell death mechanisms in the setting of diabetes-induced cardiac dysfunction. The cGAS-STING signaling pathway, which is a fundamental component of PANoptosis, has been linked to cardiac pyroptosis and hypertrophy in DCM mice, implying a critical involvement in disease development [137]. Furthermore, it has been demonstrated that the involvement of ADAM17, a protein that controls PANoptosis, improves left ventricular remodeling and function in DCM, highlighting the potential therapeutic advantages of targeting PANoptosis-related pathways [149]. Moreover, the pathophysiology of DCM as a whole, including endothelial dysfunction and metabolic abnormalities, is directly connected to the deregulation of cell death pathways, including those implicated in PANoptosis [150]. This emphasizes the importance of PANoptosis in the formation and progression of DCM, providing fresh insights into prospective therapeutic techniques that might reduce diabetes’s influence on heart health.

## Discussion

### Summarizing existing research

IFI16, a key player in DNA damage, viral infection, and inflammation, may become a central regulator of PANoptosis in heart diseases. Its expression in heart tissues suggests its involvement in disease pathogenesis. IFI16’s intricate relationship with cell death pathways adds complexity to its role in heart disease. Its functions in heart contexts involve orchestrating cellular responses to stress, shaping inflammation, and potentially influencing cell survival or demise. For instance, in MI, increased IFI16 expression correlates with enhanced inflammatory cytokine production, suggesting a direct role in exacerbating tissue damage. Furthermore, IFI16’s interactions with key cell death mediators like caspases and RIPK1 hint at a complex regulatory network influencing cardiomyocyte survival. The subtleties of this regulation could explain the variability in patient outcomes and disease manifestations.

### Proposing future research directions

Subsequent investigations ought to examine the molecular intricacies of IFI16’s involvement in PANoptosis in heart diseases. This includes:

- Clarifying how IFI16 interacts with certain inflammatory mediators during cardiac stress and charting the cellular consequences that follow.
- Examining the manipulation of IFI16’s activity as a potential therapeutic approach, such as employing small molecule inhibitors, may help lessen pathologic cell death in long-term ailments like HF.
- Analyzing the expression patterns of IFI16 at various cardiac disease stages and severity levels may provide prognostic indicators or treatment windows.
- Investigating IFI16’s function in post-injury cardiac remodeling may lead to the development of fresh tactics for halting maladaptive fibrosis and preserving heart function.
- A complete knowledge of IFI16’s downstream pathways is necessary to develop treatments that target it; these may include innovative drug delivery methods or gene therapy techniques to alter IFI16’s expression or activity in cardiac tissues.

## Conclusion

The complex biology of heart disease can be better understood by utilizing IFI16’s role in PANoptosis pathways. It might be a target for therapeutic intervention due to its dual function in controlling inflammation and cell death. The current study supports a new paradigm in therapeutic approaches by highlighting the significance of IFI16 in cardiac diseases. We have the chance to develop novel approaches that might significantly change the therapeutic management of cardiac disease by focusing on IFI16-mediated pathways, providing hope for better patient outcomes and quality of life.
